# Overcoming antifungal resistance in *Candida albicans via* RNA interference: a therapeutic perspective

**DOI:** 10.3389/fcimb.2025.1675921

**Published:** 2025-11-20

**Authors:** Akshay Kisan Mundhe, Reena Rajkumari

**Affiliations:** Department of Integrative Biology, School of Bio Sciences and Technology, Vellore Institute of Technology, Vellore, Tamil Nadu, India

**Keywords:** antifungal resistance, ergosterol biosynthesis, gene silencing, multidrug efflux pumps, nanoparticle delivery systems, RNA interference

## Abstract

*Candida albicans* remains a significant cause of nosocomial infections, with increasing antifungal resistance posing a global health challenge. Current therapies, including azoles, echinocandins, and polyenes, are increasingly limited by evolving resistance mechanisms such as efflux pump overexpression and ergosterol pathway mutations. This review explores the potential of RNA interference (RNAi) as a novel therapeutic strategy against drug-resistant *C. albicans.* While RNAi has shown efficacy in other fungal pathogens, its application in *C. albicans* is still in early stages. We discuss established antifungal targets, introduce emerging RNAi-based gene silencing approaches, and highlight potential molecular targets including *ERG1*, *ERG6*, *ERG11*, *CDR1*, *CDR2*, *CLB2*, and *GSC1*. RNAi offers a transformative route to overcome resistance at the transcriptional level, bypassing post-translational mutation-related failures of conventional drugs. With advances in small-interfering RNA (siRNA) design, chemical modifications, and nanoparticle delivery systems, RNA-based therapeutics may reshape the future of antifungal treatment.

## 1 Introduction

Candidiasis is induced by many strains of *Candida albicans*, a dimorphic fungus that resides as a commensal organism on human skin, in the oral cavity, and throughout the gastrointestinal system. *C. albicans* displays yeast and hyphal forms, together with a transitional pseudohyphal form, prompting its classification as polymorphic. This opportunistic fungus can penetrate mucosal barriers and induce infection in immunocompromised persons or those receiving extended antibiotic therapy (Ford et al., 2015; Costa-de-Oliveira and Rodrigues, 2020). Distinctively, *Candida* functions as an endogenous pathogen, frequently resulting in two principal disease categories: non-invasive and invasive candidiasis. When *C. albicans* infiltrates the circulation, it leads to candidemia, a grave illness that can spread to internal organs including the kidneys, liver, lungs, brain, eyes, and heart resulting in significant damage (Costa-de-Oliveira and Rodrigues, 2020). Of the over 15 species associated with invasive candidiasis, *C. albicans*, *C. glabrata*, *C. parapsilosis*, *C. tropicalis*, and *C. krusei* are the most clinically relevant, with *C. albicans* being the primary isolate in candidemia instances (Falagas et al., 2010; Maheronnaghsh et al., 2020; Rodriguez et al., 2020). The global morbidity and death rates linked to candidemia are significantly elevated, ranging from 40% to 50% (Cornely et al., 2020; Tsay et al., 2020; Espinel-Ingroff et al., 2021).

*Candida* is currently acknowledged as the fourth most prevalent cause of nosocomial bloodstream infections in the United States (Ford et al., 2015; Tsay et al., 2020; Nagata et al., 2021). Throughout the COVID-19 pandemic, the prevalence of *C. albicans* infections markedly escalated due to compromised host immunity, including documented instances of mucormycosis and aspergillosis in clinical environments (Leitão et al., 2016; Aldardeer et al., 2020; Ghosh et al., 2021; Nucci et al., 2021). Candidemia has thus become an increasing concern in contemporary healthcare systems.

Polymicrobial bloodstream infections are prevalent, with *C. albicans* being co-isolated alongside Staphylococcus epidermidis and *Staphylococcus aureus*. About 25% of candidemia patients are polymicrobial, highlighting the clinical intricacy of managing these infections (Carolus et al., 2019). Epidemiological studies indicate that over 75% of women encounter candidiasis at least once in their lives, while about 45% endure recurring infections (Faraji et al., 2011).

The virulence of *C. albicans* is contingent upon its morphological shape. Hyphal forms elicit more robust cytokine responses in epithelial cells than yeast forms, but certain studies indicate that yeast cells promote more production of IL-1, IL-12, and IL-12p70 in PBMCs and murine lymphocytes (Mukaremera et al., 2017). The hyphal form has increased invasiveness during the early phases of bloodstream infection, whereas the yeast form presents a heightened risk in immunocompromised individuals (Jacobsen et al., 2012). Consequently, successful therapy must address both morphological variants. *C. albicans* is sometimes referred to as a "hidden killer" due to its significant clinical effect, which is equivalent to or even exceeds that of illnesses such as TB and malaria. The rise of antifungal resistance underscores the need for new treatment techniques (Ivanov et al., 2020; Kumar et al., 2020).

A notable method is RNAi, a conserved gene-silencing process present in almost all eukaryotes. RNA interference (RNAi) modulates gene expression by post-transcriptional gene silencing. The inaugural microRNA was discovered in *Caenorhabditis elegans* by Victor Ambros in 1993 (Drury et al., 2017), succeeded by the elucidation of the RNAi process by Andrew Fire and Craig Mello in 1998—an accomplishment that garnered them the 2006 Nobel Prize in Physiology or Medicine (Fire et al., 1998; Bernards, 2006). Consequently, during the past 20 years, RNAi has attracted a lot of interest from scientists all around the world. Victor Ambros and Gary Ruvkun got their credit for the discovery of microRNA for the first time in 1993; they jointly won the Nobel Prize award in physiology or medicine (2024). In *C. albicans*, fundamental RNAi-component genes, such as Dicer-like proteins (*Dcr1*) and Argonaute (*Ago1*), have been initially identified by Drinnenberg et al. (2009) but failed to establish significant gene silencing in this species (Drinnenberg et al., 2009). A contributing factor to this failure, as indicated by recent studies by Iracane et al. (2024) (Iracane et al., 2024), is that the utilized reference strain SC5314 possesses a missense (loss-of-function) mutation in its Argonaute (*Ago1*) locus, which renders RNA interference inactive in that genetic context. Conversely, Iracane et al. (2024) demonstrated that numerous *C. albicans* isolates possess a canonical, functional *Ago1*. By integrating small RNA sequencing, long RNA profiling, and seamless CRISPR/Cas9 editing, they furnished direct evidence of an active RNAi pathway in these isolates, exemplified by the repression of subtelomeric gene families (Fire et al., 1998; Schmittgen et al., 2008; Drinnenberg et al., 2009; O'Brien et al., 2018; Lax et al., 2020; Iracane et al., 2024), analogous to the generally conserved glycolytic pathway (Leitão et al., 2016).

RNAi starts with the generation of a double-stranded RNA (dsRNA) precursor, about 90 base pairs in length. Dicer, a type III endonuclease, converts this into small-interfering RNA (siRNA), often 19–25 nucleotides in length. One strand (sense) is destroyed, while the antisense strand is integrated into the RNA-induced silencing complex (RISC) with Argonaute (Fire et al., 1998; Schmittgen et al., 2008; Moazeni et al., 2012a; Dhingra, 2020). This complex either destroys the target mRNA or inhibits its translation, contingent upon the level of complementarity.

**Figure 1:**
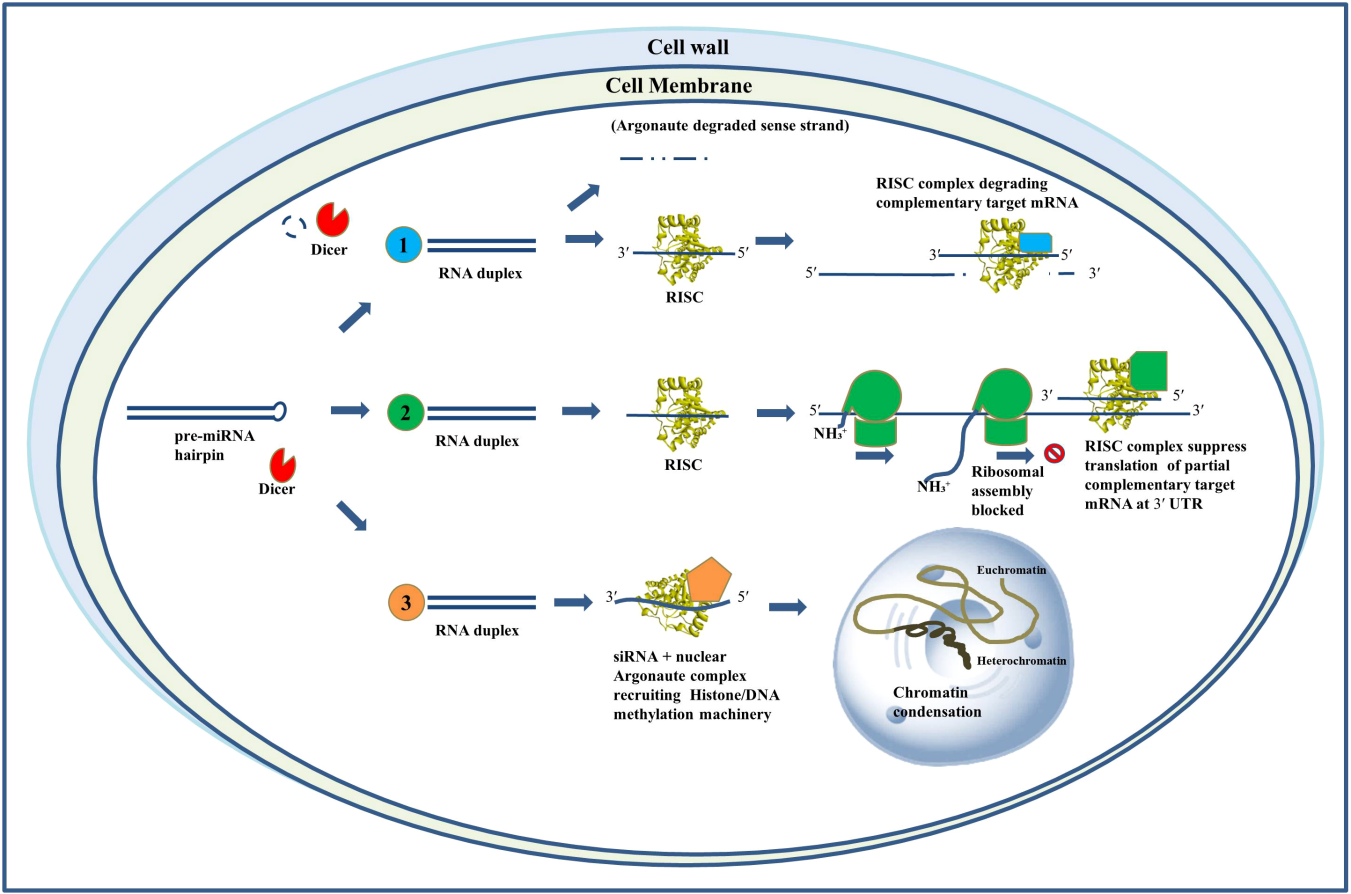
Mechanisms of action of RNA interference. Schematic representation of RNAi mechanisms: (1) siRNA-mediated mRNA cleavage via RISC, (2) miRNA-mediated translational repression through partial complementarity at the 3′ UTR, and (3) siRNA-directed transcriptional gene silencing via chromatin modification.

Investigations into miRNA and siRNA have proliferated significantly during the last twenty years, presenting transformative prospects as gene-specific therapies (Croston et al., 2018). Considering *C. albicans* medication resistance and pathogenic adaptability, RNAi presents an innovative, tailored antifungal approach. This study emphasizes RNAi's potential as a molecular instrument against drug-resistant *C. albicans*, facilitating the development of next-generation antifungal medicines. **Figure 1** illustrates the RNAi's mode of operation.

## 2 Prevalence of *Candida* infections

*C. albicans* is responsible for approximately 700,000 invasive and 2,000,000 noninvasive cases of oral candidiasis each year, and the mortality rate was recorded higher than 70% in some literature studies (Ivanov et al., 2020). More than 1.5 million individuals die each year from fungus-related illnesses, which affect over a billion people in the world. Other health problems can also lead to serious fungal infections like asthma, AIDS, cancer, organ transplantation, and corticosteroid therapies are just a few of the issues that people face. Antifungal therapy can be started right away if a correct diagnosis is made early; however, this is typically not done or is inaccessible, resulting in death, severe chronic sickness, or blindness.

**Figure 2:**
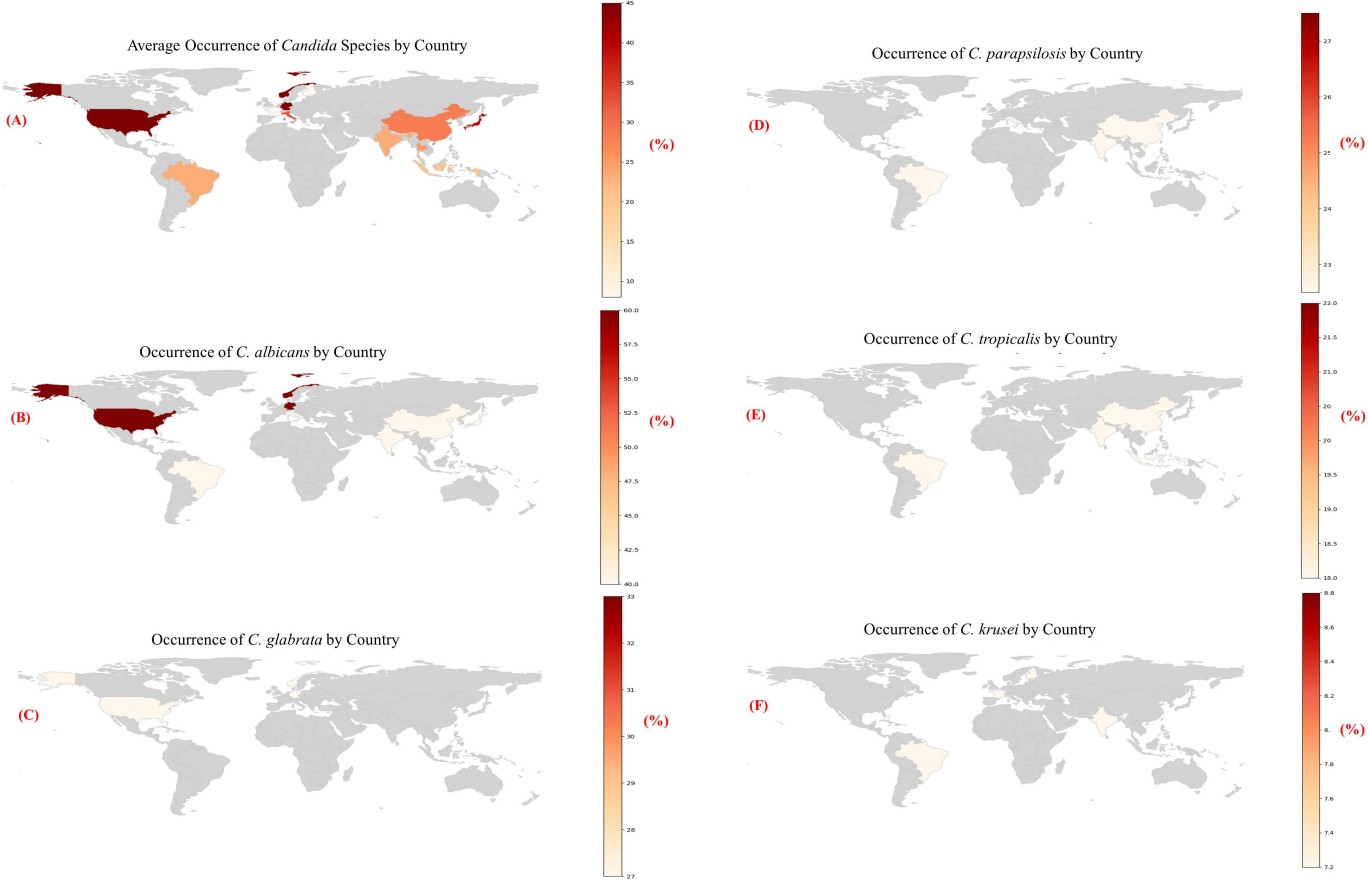
**(A-F)** Heatmaps showing the estimated occurrence of major *Candida* species by country, based on region-specific literature trends [1].

From 2013 onwards, the Leading International Fungal Education (LIFE) platform has made it easier to estimate the burden of major fungal infections country by country for more than 5.7 billion people (more than 80% of the global population). These investigations revealed that the worldwide burden varies with different countries, within countries, and also among the at-risk groups we have identified (Bongomin et al., 2017). *C. albicans* has evolved the ability to colonize multiple host niches around the world and remain there indefinitely. The environment has selected both the commensal as well as pathogenic characteristics of the yeast (Chow et al., 2021). Numerous *C. albicans* isolates were found in North and Central Europe, as well as the United States; However, non-*C. albicans* species were more common in South America, Asian countries, and Southern Europe. A detailed examination of the distribution of *Candida* species confirmed this (Falagas et al., 2010). The global geographic distribution of *Candida* species is shown in **Table 1**, **Figure 2**.

**Table 1:** Geographical occurrence of *Candida* species over the world (Falagas et al., 2010).

*Candida* Species	Geographical Region	Occurrence (%)
*C. albicans*	North Europe	>60
	Switzerland	>60
	USA	45–58
	Central Europe	45–58
	South Europe	45–58
	Asia	40–42
	South America	40–42
*C. glabrata*	USA	18.8–24
	UK	22.7
	Brazil	4.9
	Kuwait	5.6
*C. parapsilosis*	Kuwait	30.6
	South America	20.5–21.3
	Spain	23
	Australia	19.9
*C. tropicalis*	South America	20.9–24.2
	Taiwan	22.4
	USA	11–12
	Central Europe	~4
	North Europe	~4
*C. krusei*	Finland	8.5
	France	10.6

## 3 Existing treatments for candidiasis

**Figure 3:**
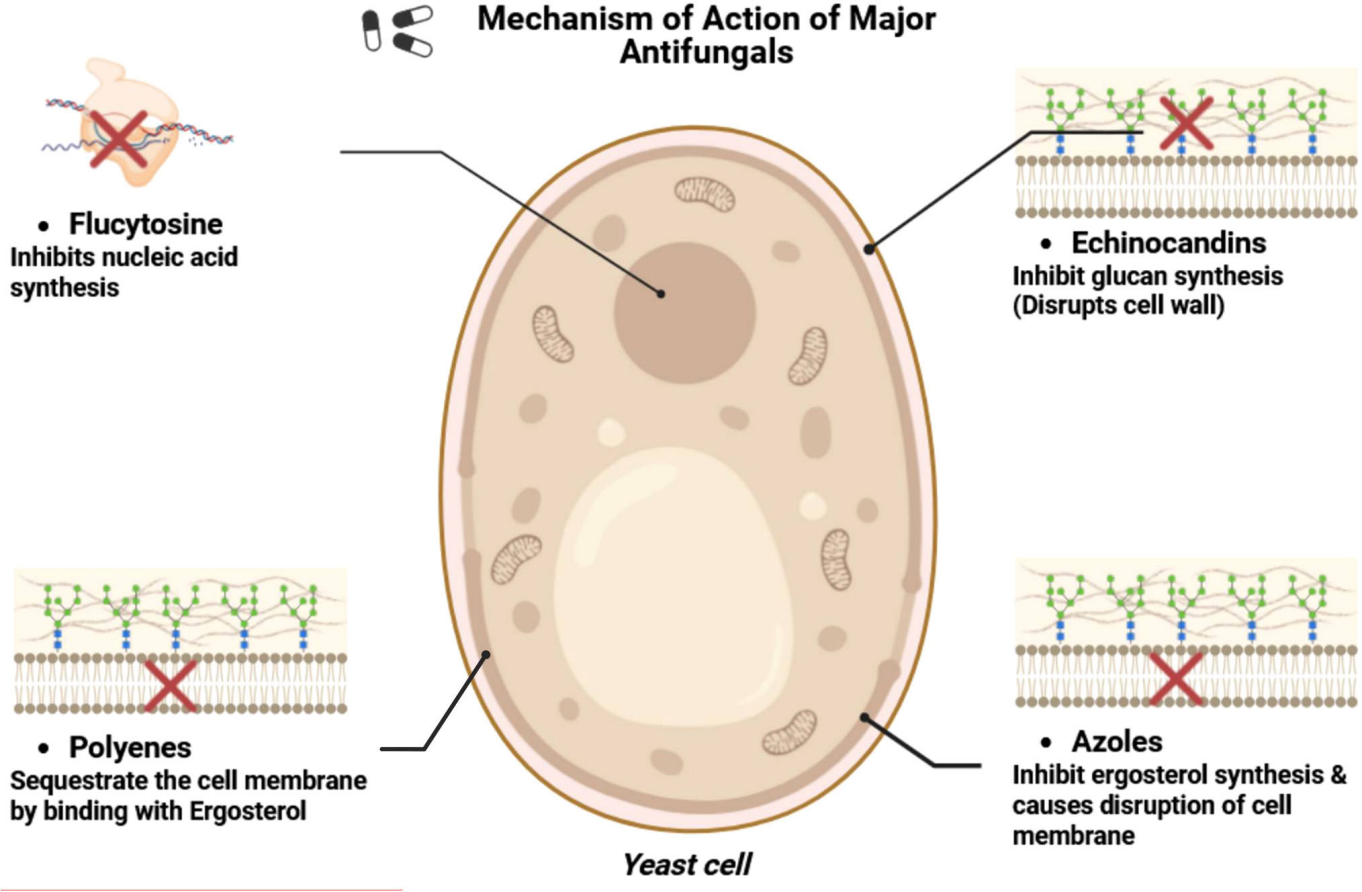
Mechanism of action of major antifungal drugs.

The existing therapy options for invasive fungal infections comprise four main types of antifungal agents: echinocandins, azoles, polyenes, and flucytosine. These medications are extensively utilized for the management of candidiasis induced by *C. albicans*, in addition to infections resulting from other pathogenic fungi such as *Aspergillus* (aspergillosis) and *Cryptococcus* (cryptococcosis). Each category of antifungals targets certain cellular processes or structures vital for fungus survival and pathogenicity. **Table 2**, **Figure 3** provide a schematic overview of the mechanisms of action of major antifungal drugs.

**Table 2:** Available major antifungal drugs and their mechanism of action.

Available antifungal drugs for treatment	Mechanism of action
Echinocandins	Inhibit biosynthesis of cell wall units, i.e., ß-1, 3-D-glucans by targeting ß-1, 3-D-glucans synthase
Azoles	Targets Lanosterol 14 α-demethylase that inhibits biosynthesis pathway of ergosterol
Polyenes	Membrane sequestration by binding with ergosterol on membrane
Flucytosine	Acts at nucleus and stops nucleic acid biosynthesis

Mode of action:

Echinocandins obstruct the production of β-1,3-D-glucan, a crucial structural polysaccharide in the fungal cell wall. Echinocandins undermine cell wall integrity by targeting the enzyme β-1,3-glucan synthase, resulting in osmotic instability and subsequent fungal cell death. This class is especially efficacious against *Candida* spp., encompassing drug-resistant variants (Ning et al., 2023).

Azoles disrupt the ergosterol production pathway, an essential element of the fungal cell membrane. They particularly block the enzyme lanosterol 14α-demethylase, a cytochrome P450-dependent enzyme that catalyzes a crucial step in ergosterol synthesis within the endoplasmic reticulum. Interference with ergosterol production leads to compromised membrane functionality, heightened membrane permeability, and suppressed fungal proliferation (Ahmed et al., 2021).

Polyenes, such as amphotericin B, exhibit antifungal activity primarily *via* binding to ergosterol in the fungal cell membrane, resulting in the formation of transmembrane pores that induce ion leakage and cell death. However, increasing information suggests that amphotericin B also demonstrates fungicidal properties by inducing oxidative stress, resulting in the production of reactive oxygen species (ROS), mitochondrial dysfunction, and lipid peroxidation. This oxidative damage significantly enhances its total antifungal efficacy, supplementing the ergosterol-binding mechanism (Yan et al., 2022). The integration of pore formation and ROS-mediated stress offers a more thorough understanding of amphotericin B's mode of action. In contrast to azoles, polyenes do not impede ergosterol production but need its presence for efficacy.

Flucytosine is a synthetic fluorinated pyrimidine derivative that interferes with nucleic acid synthesis. Upon entering fungal cells, flucytosine is transformed into 5-fluorouracil, which is then metabolized into active forms that impede both DNA and RNA production. Flucytosine is frequently utilized in combination therapy due to the fast emergence of resistance, particularly in the management of Cryptococcal meningitis and invasive candidiasis (Sigera and Denning, 2023).

Azoles and polyenes are generally not employed in combination therapy, as azoles impede the formation of ergosterol, a crucial component of fungal membranes and the major target of polyenes. This adversarial contact constrains their collective effectiveness. Echinocandins are frequently utilized in conjunction with azoles, polyenes or flucytosine, providing an expanded range of antifungal efficacy. Nonetheless, the aforementioned antifungal drugs have significant limitations, including diminished effectiveness resulting from the emergence of resistance mechanisms in pathogens (Wall and Lopez-Ribot, 2020). To overcome this problem, there are many drugs under research that will be discussed further.

The most often utilized topical antifungal medications include polyenes like nystatin and amphotericin B, as well as azoles such as miconazole and clotrimazole. Nystatin and miconazole are effective antifungals; but, extended treatment is frequently required for full cure of the illness. Miconazole, despite its more convenient forms, presents a risk of drug–drug interactions that must be meticulously evaluated during treatment. Amphotericin B and clotrimazole are excellent topical treatments for oral candidiasis; however, their restricted availability constrains broad application. In instances of oral candidiasis unresponsive to topical therapy, systemic treatment with oral fluconazole proves to be extremely efficacious. Alternative systemic agents, such as itraconazole, voriconazole, and posaconazole, are accessible in both oral and intravenous forms; however, their utilization is infrequent due to expense, adverse effects, or restrictions in their range of activity. Echinocandins, including anidulafungin and caspofungin, constitute a contemporary class of antifungal agents; however their use is restricted to intravenous administration. Isavuconazole, a novel drug, is accessible in both oral and intravenous formulations and is sanctioned for the management of invasive candidiasis. The therapy of oral candidiasis generally requires the use of nystatin, miconazole, or fluconazole. Furthermore, innovative treatment approaches, such as the formulation of new pharmaceuticals like ibrexafungerp, alongside the use of monoclonal antibodies, cytokines, and antimicrobial peptides, have significant prospects for addressing drug-resistant *Candida* infections (Quindós et al., 2019). Furthermore, RNAi has surfaced as a promising future therapy, providing a gene-specific method to silence critical fungal genes, potentially circumventing resistance mechanisms and improving treatment success.

## 4 Drug resistance and causes

Resistance to antifungal medications can be categorized as microbiological or clinical. As determined by *in vitro* susceptibility testing and compared to other isolates of the same species, microbiological resistance is described as the overcoming ability of a fungal pathogen to an antifungal treatment. It can also be classified as inherent or acquired. Unlike acquired resistance, which forms in previously susceptible fungal strains after drug administration and frequently happens because of changed gene expression, intrinsic resistance occurs naturally in some fungal strains without prior drug exposure. Contrarily, clinical resistance is the continuation of a fungal infection despite adequate treatments.

### 4.1 Resistance to azoles

The most popular antifungals are effective against various *Candida* species. A good example of this is the high (78.3%) global resistance rate of *C. krusei* to fluconazole due to its inherent resistance to the drug. Comparing *C. glabrata* to other types of *Candida*, it shows a lower dose-dependent susceptibility and a higher worldwide resistance rate (15.7%). Rarely do *C. albicans*, *C. parapsilosis*, and *C. tropicalis* display fluconazole's primary resistance (1.4%, 3.6%, and 4.1%, respectively).

Causes: These are the three main factors that contribute to azole resistance. (1) Expression of the ABC transporter family of multidrug efflux pumps confers resistance to several azoles, while expression of a key facilitator transporter confers resistance to fluconazole by lowering drug concentrations in the fungal cell. (2) Lanosterol 14-α-demethylase is changed or increased due to point mutation in *ERG11* (Whaley et al., 2017), resistance is introduced because azole binding to the enzyme site is blocked. (3) A loss-of-function mutation in *ERG3* confers resistance by stopping the hazard sterol 14-α-methyl-3,6-diol from accumulating as a result of the azole's suppression of lanosterol 14-α-sterol demethylase (Sanguinetti et al., 2015).

### 4.2 Resistance to polyenes

*Candida* species with different natural membrane sterol profiles, such as the *C. haemulonii* species complex, exhibit varying susceptibilities to polyenes in comparison to *C. albicans*. Interestingly, *C. auris* has a higher prevalence of amphotericin B resistance, with about 30% of reported isolates showing decreased polyene susceptibility.

Causes: Amphotericin B resistance is typically linked to changes in the ergosterol biosynthesis pathway as a result of loss of function (LOF) mutations in specific genes encoding important sterol synthesis enzymes. For instance, coupled loss of *ERG3* and *ERG11* activity in *C. albicans* results in ergosterol depletion, which prevents polyenes from attaching to their target (Carolus et al., 2020; Bohner et al., 2022). *ERG11* and *ERG5*, *ERG6* and *ERG2*, and *ERG6* and *ERG2* coupled modification also cause amphotericin B resistance in *C. albicans* and other *Candida* species (Bohner et al., 2022).

### 4.3 Resistance to echinocandins

Echinocandins have become the recommended therapy for invasive candidiasis (IC), overtaking azoles due to the increase in azole resistance (Sah et al., 2021). Although *C. albicans* is the predominant cause of invasive candidiasis, *C. parapsilosis* has swiftly gained in clinical significance and, in certain areas, has even eclipsed *C. albicans* as a primary pathogen. Echinocandin resistance in *Candida* species has traditionally been uncommon; nevertheless, it has been seen in *C. glabrata* and, more recently, in *C. parapsilosis*, where the incidence of resistant isolates is rising, presenting a significant treatment challenge (Tóth et al., 2019; Sah et al., 2021). In *C. albicans*, resistance remains relatively rare but is clinically important when it occurs, particularly in chronic infections subjected to prolonged echinocandin treatment (Sah et al., 2021; Ning et al., 2023).

Causes: Echinocandin resistance in *Candida* species is attributed to mutations in the *FKS* genes, which encode the catalytic portion of the 1,3-β-glucan synthase complex, the principal drug target. In *C. albicans*, these mutations aggregate in two highly conserved "hotspot" areas of *Fks1*, namely HS1 (residues 641–649) and HS2 (residues 1,345–1,365). Alterations in these areas result in significant elevations in minimum inhibitory concentration (MIC) values and may diminish glucan synthase sensitivity by as much as 3,000-fold (Sah et al., 2021). In *C. parapsilosis*, the relevant hotspots are situated at amino acid positions 652–660 (HS1) and 1369–1376 (HS2). This species demonstrates inherently elevated MIC values against echinocandins, largely attributable to the P660A alteration within *Fks1*-HS1. This substitution, while not conferring resistance on its own, contributes to the diminished baseline sensitivity of *C. parapsilosis* and elucidates its increased tendency to develop echinocandin resistance under clinical strain (Davari et al., 2020; Ning et al., 2023).

### 4.4 Resistance to flucytosine

In 2020, Ezeadila evaluated 88 isolates for antifungal activity, and the results revealed that none of the isolates of *C. albicans, C. krusei*, or *C. glabrata* were susceptible to flucytosine. In the UK, 80% of *C. albicans* were discovered to be resistant, according to Hope (2004). The results may vary depending on the method used to gauge the isolates' susceptibility. The disc approach has been proposed to evaluate *C. albicans* flucytosine sensitivity (Hope et al., 2004). This method has been discovered to be sensitive, though not always exact. For *C. tropicalis* and *C. parapsilosis*, respectively, a very low susceptibility of 4% and 2% was discovered (Hope et al., 2004; Ezeadila et al., 2020).

Causes: According to the study by Dodgson (2004), a point mutation in the *C. albicans* gene uracil phosphoribosyltransferase (*FUR1*) leads to the substitution of thymine for cytosine at nucleotide position 301. This mutation significantly influences the susceptibility of *C. albicans* strains to 5-fluorocytosine (5FC), an antifungal pyrimidine analogue. The minimal inhibitory concentrations (MICs) of 5FC were reported as approximately 0.25 μg/ml in strains lacking the mutant allele, 0.5 μg/ml in strains carrying a single copy of the mutant allele, and up to 16 μg/ml in strains harboring two copies of the mutant allele. The presence of this mutant allele confers resistance to 5FC and represents the principal genetic basis for antifungal resistance in *C. albicans.* Notably, this was the first report describing a genetic mutation responsible for 5FC resistance in this pathogenic yeast (Dodgson et al., 2004).

This study elucidates antifungal resistance mechanisms in both *C. albicans* and non-albicans *Candida* species, offering a comparative viewpoint to underscore the distinctions and commonalities in resistance and pathogenicity characteristics. We do not assert that RNAi is functionally operative in all *Candida* species. RNAi activity has been confirmed solely in specific isolates of *C. albicans* (Iracane et al., 2024) (Iracane et al., 2024), while *C. parapsilosis* and *C. tropicalis* have yet to exhibit a functional RNAi pathway. Additionally, *C. glabrata* and *C. auris* are regarded as RNAi-inactive due to the lack of canonical components (Drinnenberg et al., 2009). The emphasis of RNAi-based therapy techniques is on *C. albicans*, with non-albicans species included solely for comparative resistance analysis.

## 5 Executing new drugs in research

A newly found chemical, a herbal remedy, or a drug that functions effectively when paired with other chemical compounds can all be considered novel antifungal agents (Chiou et al., 2000; Ruiz-Baca et al., 2021). Flavonoids like luteolin, quercetin & quercetin glycoside, quercitrin-isoquercitrin, and others have been examined as supplementary antifungal drugs, however most of them exhibit some cytotoxicity, and attempts are being undertaken to reduce the toxicity. Some of those might later turn out to be effective fungicidal medicines (Ivanov et al., 2020). Compared to the *C. albicans* ATCC 10231 biofilm, planktonic *C. albicans* are more susceptible to cumin extract. According to research by Hartini et al. (2019), Cuminaldehyde crosses the cell membrane to enter the cell and obstructs cell production (Hartini et al., 2019). The **Table 3** denotes some of the new compounds that are under the clinical research trial for the treatment of *C. albicans* (Ruiz-Baca et al., 2021).

**Table 3:** New compounds under clinical research for the treatment of *Candida* infections.

Source	Compound	Target	Mechanism of action	Citation
Synthetic compounds	Rezafungin (CD101)	β-d-glucan	β-d-glucan synthase inhibition	(Garcia-Effron, 2020; Ruiz-Baca et al., 2021)
	Ibrexafungerp (SCY-078)	β-d-glucan	β-glucan synthase inhibition	(Schell et al., 2017; Ruiz-Baca et al., 2021; Tagirova et al., 2024)
	VT-1161	Ergosterol	Specific for fungal *Cyp51*	(Warrilow et al., 2014; Ruiz-Baca et al., 2021)
	Fosmanogepix (APX001)	Glycosyl phosphatidylinositol	GPI biosynthesis inhibition	(Berkow and Lockhart, 2018; Ruiz-Baca et al., 2021; Hodges et al., 2023)
	Aureobasidin A	Inositol phosphorylceramide synthase	Sphingolipids inhibition	(TAKESAKO et al., 1993; Ruiz-Baca et al., 2021; Teymuri et al., 2021; Wu et al., 2025)
	Efungumab (or Mycograb)	HSP90	Antibody binds to fungal *HSP90*	(Matthews et al., 2003; Louie et al., 2011; Ruiz-Baca et al., 2021)
	Geldanamycin- like agents	HSP90	*HSP90* inhibition	(Lamoth et al., 2015; Ruiz-Baca et al., 2021)
	AR-12	Likely blocks fungal acetyl-CoA synthetase 1	Downregulation of chaperone proteins	(Koselny et al., 2016; Ruiz-Baca et al., 2021)
	T − 2307	Mitochondrial membrane potential	Respiratory chain complexes inhibition	(Yamashita et al., 2019; Ruiz-Baca et al., 2021)
	VL-2397 (ASP2397)	Unknown	Unknown, but taken up by Sitl	(Dietl et al., 2019; Ruiz-Baca et al., 2021)
Repurposed compounds	Rifampin	RNA polymerase	Enhance the antifungal activity	(Christenson et al., 1987; Ghannoum and Rice, 1999; Ruiz-Baca et al., 2021)
	Verapamil	Calcium channel	Enhance the antifungal activity	(Liu et al., 2016; Ruiz-Baca et al., 2021)
Antibiotic peptides	Lysozyme	Secreted aspartic protease (SAP)	Reduces *SAP* activity and secretion	(Wu et al., 1999; Gálvez-Iriqui et al., 2020; Ruiz-Baca et al., 2021)
	Lactoferrin (hl.f)	Antimicrobial activity	Produces cationic antimicrobial peptide lactoferricin	(Samaranayake et al., 1997; Fernandes and Carter, 2017; Ruiz-Baca et al., 2021)
	Human b-defensins (HBD), Cathelicidins	Cell membrane	Increases membrane permeability	(Krishnakumari et al., 2009; Ruiz-Baca et al., 2021)
	Histatin-5	Non-lytic ATP efflux	Inhibition of adhesion	(Edgerton and Koshlukova, 2000; Ruiz-Baca et al., 2021)
Medicinal plants	Scutellaria aicalensis (flavonoid baicalein)	Unknown	Induces apoptosis	(Serpa et al., 2012; Ruiz-Baca et al., 2021)
	Cymbopogon nardus (essential oils)	Unknown	Inhibits hyphal growth	(De Toledo et al., 2016; Ruiz-Baca et al., 2021)
	Artemisia judaica (essential oil)	Germination	Inhibits the germination tube and biofilms formation	(Köse et al., 2016; Ruiz-Baca et al., 2021)
Natural compounds	Thymol (terpene)	Ergosterol	Binds to ergosterol in the membrane resulting in cell death	(De Castro et al., 2015; Jung et al., 2021; Ruiz-Baca et al., 2021)
	Carvacrol (terpene)	Cell membrane	Alters cellular cytoplasmic membrane and induces apoptosis	(Dalleau et al., 2008; Ahmad et al., 2011; Lima et al., 2013; Ruiz-Baca et al., 2021)

## 6 RNA interference a new perspective for treating fungal infections

RNA interference has been successfully utilized to inhibit disease-related genes in several biological systems. This method has recently acquired importance in organ transplantation research, demonstrating potential in gene silencing of ischemia–reperfusion injury (IRI)-related and graft rejection pathways to improve organ viability and post-transplant outcomes (Brüggenwirth and Martins, 2020). In antifungal therapy, siRNA provides a fundamentally different mechanism of action than traditional medications. Traditional antifungal medicines typically target metabolic or enzymatic processes at the protein level, whereas RNAi operates earlier in the gene expression cascade, functioning at the transcriptional stage to degrade or block particular mRNA transcripts. This upstream control allows RNAi to circumvent resistance mechanisms caused by post-translational changes or point mutations that modify target enzyme conformations (Bruch et al., 2022; Ma et al., 2022; Iracane et al., 2024).

Due to these benefits, RNAi has emerged as a potential method for managing *Candida* infections, signifying a conceptual progression beyond previous small-molecule treatments. While this technique may have been unimaginable two decades prior, contemporary advancements in RNA design, delivery mechanisms, and molecular biology have rendered it progressively attainable. Consequently, RNAi-based treatments have to be seen as a supplementary enhancement to current antifungal approaches, thereby expanding the molecular framework for addressing *C. albicans* infections. Evidence from proof-of-principle investigations in plant pathogenic fungi has shown that RNA-based treatments may effectively suppress fungal virulence and proliferation, highlighting their translational potential in medical mycology (Gebremichael et al., 2021; Bruch et al., 2022).

By utilizing the CRISPR-Cas9 technique to delete the *AFR1* gene, Xiaoyu et al. (2023) verified that the enhanced ABC transporter *Afr1* in the RNAi mutants contributed to fluconazole resistance. Their research demonstrates that the RNAi pathway in *Cryptococcus neoformans* inhibits fluconazole resistance and plays a role in the metabolism of nutrients. This provides insightful information about the RNAi mechanisms in *Cryptococcus* as well as helpful recommendations for managing cryptococcosis in clinical settings (Ma et al., 2022). Researchers Mousavi et al., in 2015, wanted to know whether silencing the *cyp51A* gene helped azole-resistant *Aspergillus fumigatus* strains. Using the complementary DNA sequence of the *cyp51A* gene from *A. fumigatus*, a 21-nucleotide siRNA was assembled. The *cyp51A* gene was silenced in germinated conidia at doses of 15, 20, 25, and 50 nM, and the *cyp51A* mRNA level was measured using an RT-PCR method after azole-resistant *A. fumigatus* was grown on broth medium. At a dose of 50 nM, siRNA significantly reduced *cyp51A* gene expression (P < 0.05) after successfully transfecting hyphae using the gene-editing technology. When compared to the control cells that were not treated with siRNA, the treated cells showed a decrease in the MIC of itraconazole, going from 16 to 4 µg/ml (Mousavi et al., 2015).

**Figure 4:**
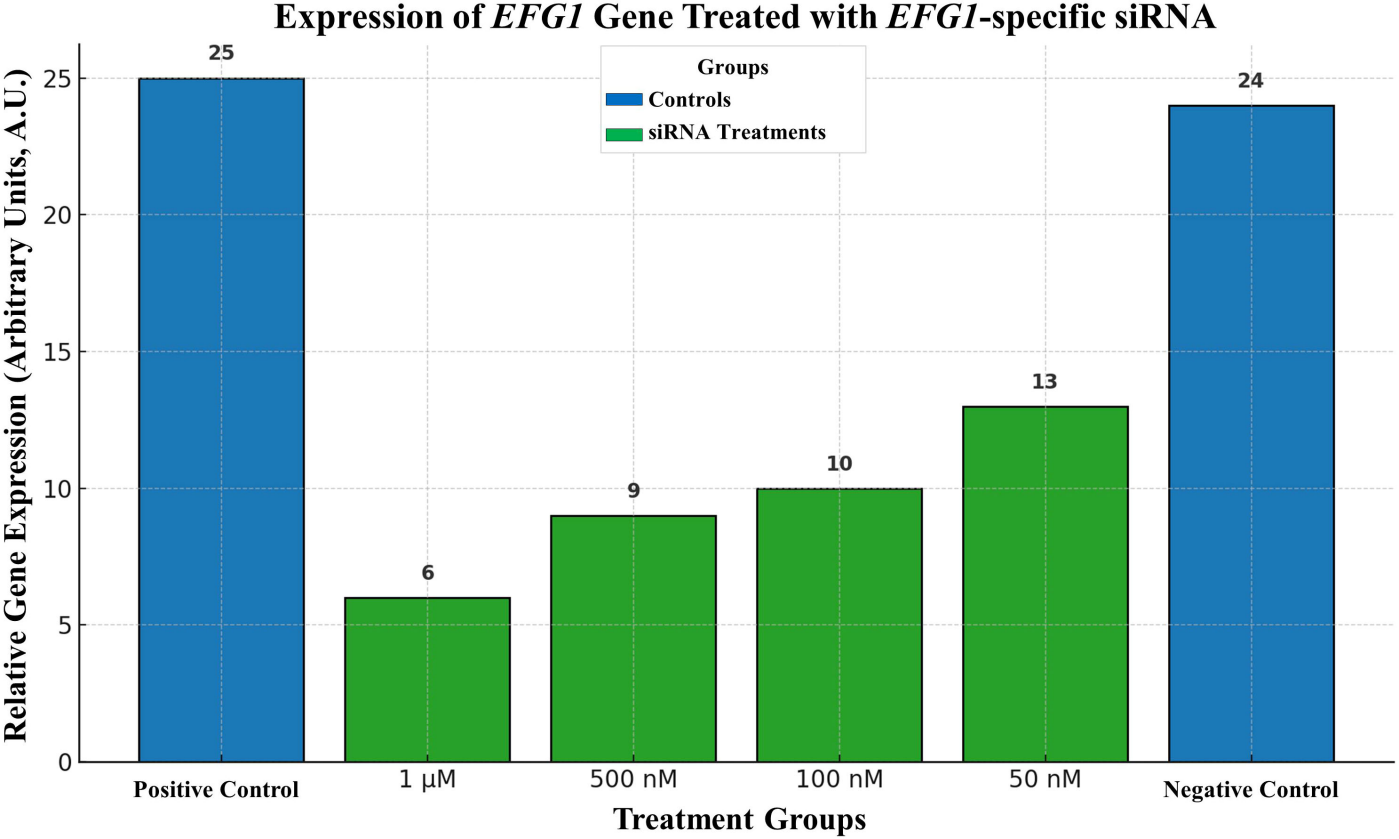
Expression of *EFG1* gene treated with *EFG1*-specific siRNA.

The Moazeni M. et al., in 2012, have tried to control *C. albicans* growth for the first-time using RNAi. Their novel experiments targeted the key regulatory gene *EFG1*. The *EFG1* gene is responsible for the formation of germ tubes in *C. albicans*. Following germ tube, the pathogen forms hyphae that are required for penetrating the host epithelial cells. It also up-regulates or enhances the expression of more downstream genes required for adhesion (e.g., *ALS1* and *ALS3*) and invagination (e.g., *SAP4*, 5, and 6) of the host cell. Moazeni M. et al., have gotten satisfactory results that can be seen in the **Figure 4**. Their research inspired us to think that RNAi would be a helpful treatment for fungal infections, and their results gave us hope (Moazeni et al., 2012a).

Bar 1) represents a collection of non-treated positive control cells., 2) 1 μM siRNA-treated cells, 3) 500 nM siRNA treated cells, 4) 100 nM siRNA-treated cells, 5) 50 nM siRNA-treated cells, and group 6) 1 μM non-related siRNA-treated negative control cells. Moazeni M. et al. (2012) (Costa-de-Oliveira and Rodrigues, 2020).

The development of RNA-based therapies against human fungal diseases faces several significant obstacles. (1) To make it easier to identify and take advantage of prospective targets, we need to better understand how RNA regulation works in human fungal infections. (2) RNA must be guided to the proper site of infection and restrain the activation of host inflammatory pathways. (3) The RNA therapy must be capable of entering the fungal cell across what is frequently a strong cell wall and must be resistant to destruction by host or pathogen-produced RNases. Even with these challenges, the numerous treatment options that have been successful in treating phytopathogenic fungi make RNA-based medicines a potential development in the management of human fungal illnesses (Bruch et al., 2022) with little modifications in the RNA molecules. Chemical modifications can extend siRNA's *in vivo* lifespan, improve cellular uptake, and improve serum stability (Brüggenwirth and Martins, 2020). Substituting the uridine with altered N1-methyl-pseudouridine for the mRNA vaccines against SARS-CoV-2 restricts the detection by host receptors specific for nucleic acid and bypasses the activation of host inflammatory pathways (Anderson et al., 2010; Karikó et al., 2012; Corbett et al., 2020); the modification to the nucleic acid backbone at 2′-O by 2′-O-methylation to minimize immunogenicity (Karikó et al., 2005; Choung et al., 2006); and improves in RNA production are able to limit production of unwanted immunological outcomes such as double-stranded RNA (Nelson et al., 2020).

The efficiency of RNAi medication depends on the penetration of the siRNA into the target cell's cytosol. The lipid nanoparticle (LNP) coated delivery of siRNA molecules to the hepatocytes has been clinically proven to be highly effective for the treatment of hypercholesterolemia and transthyretin-induced amyloidosis. LNP technology is being extended to various tissues and illnesses, and although it has not yet been clinically utilized for fungal infections, this platform possesses potential for the future development of siRNA-based treatments targeting *Candida* species (Wan et al., 2014).

When taken together, these advancements suggest that future medical practices will change for all types of illnesses, but further investigation is needed to see how they can be used to treat human fungal pathogens. From siRNA-based cancer treatments to mRNA vaccinations that offer protection against SARS-CoV-2, RNA-based therapies are proven to be transforming medicine. Opportunities to use these developments to the treatment of fungal infections in humans will present themselves as these therapeutic approaches evolve (Bruch et al., 2022). To prevent off-target effects and encourage growth halt or death of the target infection, particular targets inside the fungal pathogen must ultimately be addressed that shouldn’t have similarity with any of the host genes.

## 7 Potential targets of *Candida* for RNAi therapy

Only a few numbers of genes (such as *EFG1*, *ALS*, and *SAP* family genes) have been the targets for RNAi activity against *C. albicans* in the minimal research that has been done so far. Here, we are outlining some potential new RNAi targets for *Candida* (Moazeni et al., 2012a; Moazeni et al., 2012b; Moazeni et al., 2014; Araújo, 2021). Alongside the identification of these potential targets, it is crucial to examine their significance for fungal survival or pathogenicity upon deletion or silencing, and to evaluate how RNAi therapy might circumvent resistance mechanisms, such as point mutations, that diminish the effectiveness of traditional antifungal agents.

### 7.1 Squalene epoxidase (*ERG1*)

The gene for squalene epoxidase is encoded by *ERG1*. It is a crucial enzyme in the ergosterol biosynthesis pathway, operating upstream of lanosterol 14α-demethylase. This step is crucial for ergosterol production; deletion or significant suppression of *ERG1* results in compromised membrane integrity and decreased viability in *C. albicans*. Squalene epoxidase is inhibited by allylamines, including terbinafine and naftifine (Khodavandi et al., 2014); still, hypersensitivity responses and unpleasant effects (burning, irritation, itching) restrict their clinical application (Matpathi et al., 2023). By employing RNAi, it is possible to silence *ERG1* expression, resulting in a fatal impact on the fungus while reducing drug-related toxicity. Significantly, RNAi focuses on mRNA sequences, rendering the method less susceptible to point mutations in protein-coding areas that typically modify drug-binding sites.

### 7.2 Lanosterol 14 α demethylase (*ERG11*)

Lanosterol 14-demethylase is the key player in the ergosterol biosynthesis pathway. It is the cytochrome p450 enzyme, located in the membrane of the endoplasmic reticulum. The enzyme has been an important target for azole antifungal drugs for treating *Candida* infections (Henry et al., 2000; Ivanov et al., 2020). By inhibiting the biosynthesis pathway of ergosterol, a major cell surface component, a cell can no longer be alive. The critical importance of *ERG11* is well known; mutants lacking this gene are unable to survive. Resistance to azoles frequently develops due to point mutations in ERG11 that modify its conformation, hence diminishing drug binding (Ivanov et al., 2020). RNAi operates at the transcript level; hence, these resistance mutations fail to shield the fungus from siRNA-mediated gene silence, assuming the siRNAs are engineered to target conserved coding or untranslated areas. Consequently, RNAi provides a benefit by circumventing medication resistance processes associated with changes in protein structure.

### 7.3 Sterol 24-C-methyltransferase (*ERG6*)

Sterol 24-C-methyltransferase, encoded by *ERG6*, operates in the latter phases of ergosterol biosynthesis, facilitating the transformation of zymosterol into fecosterol. While *ERG6* is not absolutely necessary for *C. albicans* survival, its disruption modifies sterol composition and membrane architecture, resulting in diminished virulence and a decreased capacity to colonize the host (Jacko et al., 2025). No clinically used drugs targeting *ERG6* have been discovered so far, rendering RNAi a unique method to limit its expression. Inhibiting *ERG6* may compromise the fungal membrane and enhance host immune elimination. The selectivity of RNAi (Leung and Whittaker, 2005) enables the precise targeting of *Candida* sequences while preserving host sterol pathways (Moazeni et al., 2012a).

### 7.4 Drug efflux ABC type transporter (*CDR1* & *CDR2*)

*CDR1* and *CDR2* are abbreviations for *Candida* drug resistance. The ABC-type transporters are the drug efflux membrane transporters of *C. albicans*. The overexpression of the *CDR1* and 2 has been observed with increasing doses of available antifungal drugs on the market like azoles and polyenes (Prasad and Rawal, 2014; Ivanov et al., 2020). Their overexpression is often noted in drug-resistant clinical isolates, rendering them appealing targets for RNAi. Gene knockdown can reinstate sensitivity to antifungal drugs. In drug-resistant isolates exhibiting *CDR* overexpression, the combination of azoles with siRNA-mediated silencing of *CDR* genes may yield synergistic benefits by diminishing efflux and augmenting intracellular drug accumulation.

### 7.5 1, 3-beta-glucan synthase (*GSC1*)

Glucan synthase, partially expressed by the FKS gene family, is responsible for the synthesis of β-1,3-glucan, a vital component of the cell wall. This enzyme is essential for the vitality of fungal cells. Echinocandin antifungals (e.g., caspofungin, micafungin, anidulafungin) inhibit glucan synthase; nonetheless, clinical resistance often develops owing to mutations in critical areas of *FKS* genes. These mutations reduce drug binding while maintaining enzyme function (Davari et al., 2020; Sah et al., 2021). RNAi, however, inhibits protein production at the mRNA level, bypassing resistance linked to modified protein structure. Silencing *GSC1* by RNAi might completely inhibit cell wall manufacturing, resulting in fungal mortality.

### 7.6 Cell cycle regulatory gene (*CLB2*)

The *CLB2* gene encodes a B-type cyclin essential for cell cycle progression in *C. albicans*, especially during late anaphase. Loss-of-function mutations or transcriptional suppression of *CLB2* induce cell cycle arrest during late anaphase, resulting in aberrant elongation into filamentous or hyphal structures, subsequently causing a gradual decline in viability (Richardson et al., 1992; Mendenhall and Hodge, 1998; Berman, 2006; Chow et al., 2021). The *CLB2* gene product, clb2p, forms complexes with cdc28p. This active complex breaks the spindle assembly checkpoint arrest in anaphase and progresses the cell cycle (Donovan, 2000). The role of the clb2p/cdc28 active complex can be analogous to that of the apc-20/cdh1 active complex. The *APC-20/Cdh1* complex is necessary for the degradation of the metaphase-promoting factor (*MPF*) to complete the late anaphase in higher eukaryotes. Due to its importance in cell proliferation, we can use “keystone gene” terminology for the *CLB2* gene (an analogy from ecology, i.e., keystone species) (Hallikas et al., 2021). Consequently, RNAi-induced silencing of *CLB2* is a viable antifungal approach, given that this target is unique to fungi and minimizes the risk of detrimental interactions with the host. As such, by inhibiting the *CLB2* gene, we can arrest both yeast as well as the hyphal (invasive) form of *C. albicans* in the anaphase of the cell cycle that provides powerful means of impairing fungal growth and pathogenicity.

The aforementioned possible targets differ in their essentiality, although all play a crucial role in either fungal survival (*ERG1*, *ERG11*, *GSC1*, *CLB2*) or virulence and resistance (*ERG6*, *CDR1*/*CDR2*). RNAi treatment has a distinct benefit over traditional antifungals by directly silencing transcripts, hence reducing susceptibility to resistance caused by protein point mutations (e.g., in *ERG11* or *FKS* hotspots). This transcript-level activity guarantees that even resistant alleles remain vulnerable when conserved areas are targeted. Nonetheless, meticulous siRNA design is essential to attain specificity and reduce off-target silencing. RNAi together offers a potential and versatile approach to address the shortcomings of existing antifungal treatments.

**Figure 5:**
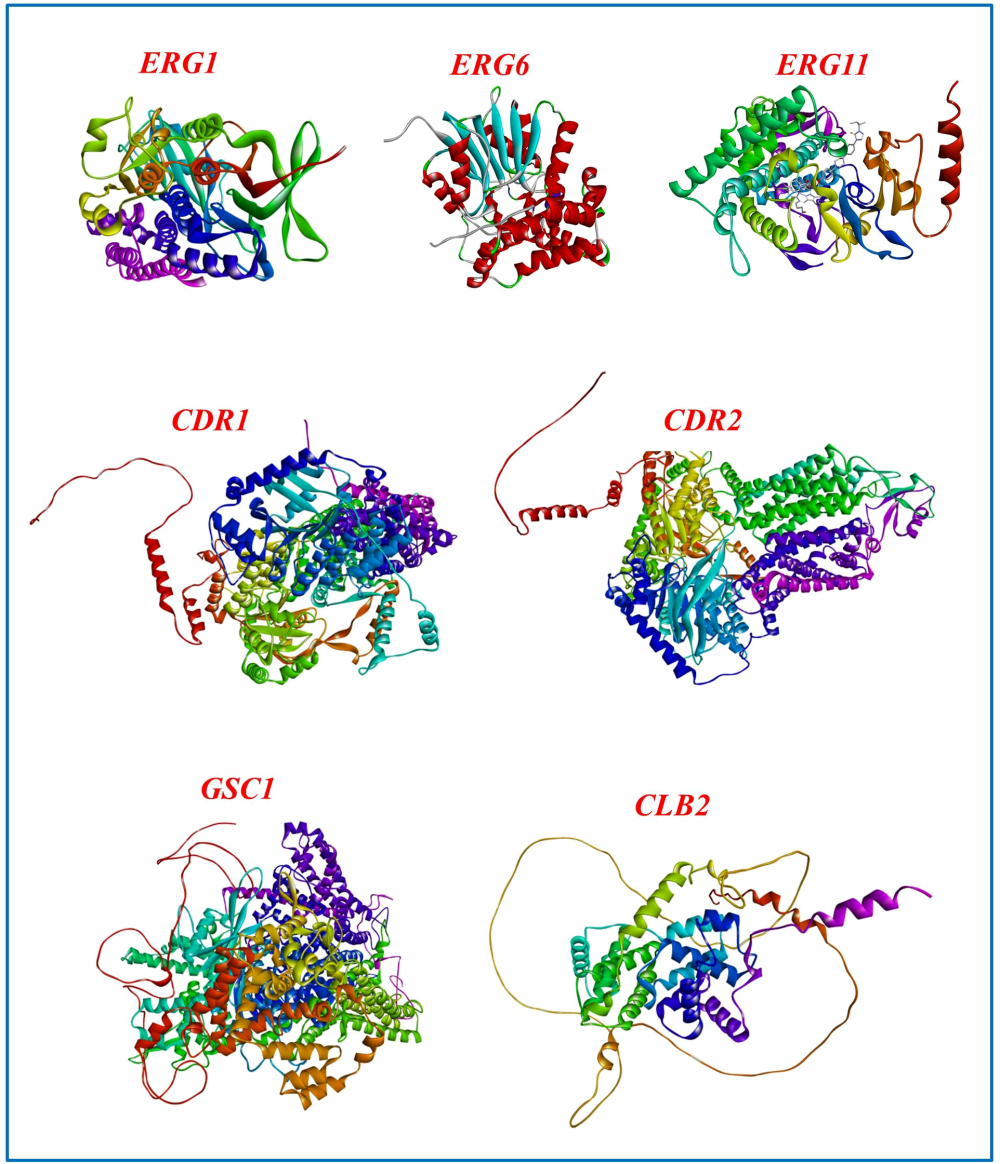
Structural models of *C. albicans* proteins suggested as RNAi targets. The experimentally determined crystal structure of *ERG11* (CYP51) complexed with itraconazole and heme (PDB ID: 5V5Z) is presented, whereas the three-dimensional structures of *ERG1*, *ERG6*, *CDR1*, *CDR2*, *GSC1*, and *CLB2* were derived from AlphaFold.

While RNAi therapies operate at the mRNA level, showcasing AlphaFold-predicted 3D structures (**Figure 5**) underscores the biological importance of these targets. Structural visualization demonstrates that these proteins are necessary, well-folded, conserved, and functionally confirmed elements of vital fungal processes. This underscores their therapeutic significance: inhibiting their transcripts would directly impair essential protein activities. Furthermore, whereas RNAi inhibits gene expression at an upstream level, understanding the downstream protein structure is essential for prospective combination treatments that may integrate RNAi with traditional small-molecule inhibitors aimed at the same protein. Consequently, the structural representation enhances the RNAi-based methodology by emphasizing the appeal and need of these gene products as antifungal targets.

Among the ergosterol biosynthesis and drug resistance-related proteins in *C. albicans* examined herein, *ERG11* (lanosterol 14-α demethylase, CYP51) is distinctive for possessing experimentally determined crystal structures, including complexes with azole antifungals (e.g., PDB IDs: 5FSA, 5TZ1 (Hargrove et al., 2017) and 5V5Z (Keniya et al., 2018)). In **Figure 5**, *ERG11* corresponds to the 5V5Z structure, which depicts the *C. albicans* CYP51 complexed with itraconazole and heme. In contrast, the structures of *ERG1, ERG6, CLB2, GSC1, CDR1*, and *CDR2* are solely accessible *via* AlphaFold predictions. This distinction is essential, as experimental models provide more assurance regarding active-site geometry and ligand interactions, whereas AlphaFold predictions are beneficial for hypothesis formulation in the absence of crystallographic evidence.

## 8 Delivery strategies and challenges

The effective translation of RNAi-based antifungal treatments relies significantly on the advancement of efficient and tailored delivery mechanisms that can penetrate the robust fungal cell wall. The structural barrier, consisting of chitin, β-glucans, and mannoproteins, significantly restricts the internalization of siRNA molecules (Gebremichael et al., 2021; Bruch et al., 2022).

Contemporary study investigates various delivery methodologies. Lipid nanoparticle (LNP) systems, derived from mRNA vaccine technologies, provide protection against RNase-mediated degradation and facilitate cellular absorption *via* endocytosis (Wan et al., 2014; Brüggenwirth and Martins, 2020; Corbett et al., 2020). Cationic polymers, including polyethyleneimine (PEI) and chitosan-based nanocarriers, augment electrostatic interactions with fungal surfaces, hence boosting penetration efficiency (Gebremichael et al., 2021). Cell-penetrating peptides (CPPs) and aptamer conjugates provide species-specific identification of fungal surface motifs, enhancing targeting accuracy and reducing host toxicity.

Despite these advancements, many critical barriers persist: maintaining specificity for fungal cells, preserving siRNA stability in serum and host environments, and averting unintended immune activation continue to pose significant hurdles (Brüggenwirth and Martins, 2020; Bruch et al., 2022). Chemical changes, including 2′-O-methylation, locked nucleic acids (LNAs), and phosphorothioate linkages, might improve resistance to nuclease degradation and diminish innate immune recognition (Karikó et al., 2005; Corbett et al., 2020; Nelson et al., 2020).

The ongoing enhancement of multifunctional nanocarriers including fungal-specific ligands and controlled-release mechanisms will be crucial for developing effective, targeted, and therapeutically applicable RNAi treatments for *C. albicans*.

## 9 Discussion

The rise of antifungal resistance in *C. albicans* poses a significant threat to world health, requiring innovative treatment approaches beyond standard antifungal medications (Ford et al., 2015; Aldardeer et al., 2020; Costa-de-Oliveira and Rodrigues, 2020; Tsay et al., 2020). RNAi offers a promising gene-specific strategy for targeting critical fungal virulence and resistance pathways by silencing transcripts at the mRNA level (Fire et al., 1998; Moazeni et al., 2012a; Bruch et al., 2022). In contrast to conventional antifungal agents, which often fail due to protein level mutation caused conformational alterations, RNAi acts upstream, reducing the likelihood of resistance mediated by altered drug binding sites (Fire et al., 1998; Bruch et al., 2022).

A primary advantage of RNAi is its high specificity. Gene silencing may be accomplished with great accuracy by creating siRNAs or shRNAs that are complementary to essential fungal mRNA transcripts, hence limiting collateral effects (Brüggenwirth and Martins, 2020; Bruch et al., 2022). The efficacy of lipid nanoparticle (LNP)-based mRNA vaccines against SARS-CoV-2 presents a persuasive model for the application of analogous platforms to administer RNAi compounds aimed at fungal infections (Karikó et al., 2012; Corbett et al., 2020). The translational implementation of RNAi treatment targeting human fungal pathogens such as *C. albicans* is still yet to develop and encounters several significant obstacles like siRNA delivery and stability.

Another aspect to contemplate is if resistance processes may compromise RNAi treatment, similar to traditional antifungals. In contrast to small-molecule medicines, which diminish efficacy due to point mutations that modify protein targets, RNAi operates at the transcript level. siRNAs exhibiting absolute complementarity facilitate mRNA cleavage, however even partial complementarity might impede translation by obstructing ribosome advancement. Consequently, RNAi possesses the benefit of circumventing several mutation-induced resistance mechanisms that undermine conventional antifungal agents. Nonetheless, meticulous siRNA design targeting conserved transcript areas is crucial to guarantee extensive efficiency across clinical isolates. Additionally, RNAi activity is not generally shown among all *C. albicans* strains. Iracane et al. (2024) established that the reference strain SC5314 is inactive in RNAi due to a faulty *AGO1* allele, but most clinical isolates retain a functional RNAi pathway. This heterogeneity presents a significant issue: individuals infected with RNAi-deficient isolates may exhibit a lack of response to RNAi treatment (Iracane et al., 2024). Pre-therapeutic evaluation for RNAi efficacy and meticulous siRNA design aimed at conserved areas are thus crucial to enhance clinical relevance.

In comparison, conventional antifungal treatments while beneficial in certain instances are progressively hindered by the emergence of resistance, off-target toxicity, and pharmacokinetic constraints. Although polyenes and azoles have been fundamental in therapeutic applications, their effectiveness is sometimes undermined by the overexpression of efflux pumps or alterations in the ergosterol biosynthetic pathway (Ford et al., 2015; Sanguinetti et al., 2015; Whaley et al., 2017; Carolus et al., 2020). Echinocandins, while efficacious, are only administered intravenously and exhibit diminished efficiency against certain species (Aldardeer et al., 2020; Tsay et al., 2020; Ning et al., 2023). RNAi, if well used in clinical settings, might provide a whole new category of antifungals that function independently of current drug resistance mechanisms.

Subsequent research should concentrate more on refining delivery vectors, improving siRNA stability, and confirming conserved, fungus-specific targets. In this context, previously identified candidate genes namely *ERG1, ERG6, ERG11* (ergosterol biosynthesis) (Henry et al., 2000; Ivanov et al., 2020), *CDR1* and *CDR2* (drug efflux) (Prasad and Rawal, 2014; Ivanov et al., 2020), *GSC1* (cell wall synthesis) (Carolus et al., 2020; Ning et al., 2023), and *CLB2* (cell cycle regulation) (Richardson et al., 1992; Mendenhall and Hodge, 1998; Berman, 2006) serve as robust foundations for therapeutic investigation, contingent upon siRNA design that considers sequence conservation among clinical isolates to reduce off-target effects. Combining RNAi strategies with current antifungal medications may enhance effectiveness, while the integration of RNAi with CRISPR technologies might broaden the scope of functional genomics and therapeutic uses (Bruch et al., 2022; Ma et al., 2022). These efforts will be essential for converting RNAi from a basic cellular process into an effective antifungal approach.

## 10 Conclusion

In brief, RNAi has significant potential as an innovative antifungal strategy through the utilization of transcript-level gene silencing. The success of this endeavor will hinge on progress in tackling siRNA instability, delivery obstacles, and diversity in RNAi activity across different strains of *C. albicans*. Ongoing advancements in RNA chemistry, nanocarrier technology, and functional genomics position RNAi as a possible complement or enhancement to current antifungal therapy for drug-resistant *C. albicans*.
